# Uncover the anticancer potential of lycorine

**DOI:** 10.1186/s13020-024-00989-9

**Published:** 2024-09-08

**Authors:** Yan-Ming Zhang, Ting Li, Chun-Cao Xu, Jia-Yu Qian, Hongwei Guo, Xiaolei Zhang, Zha-Jun Zhan, Jin-Jian Lu

**Affiliations:** 1 State Key Laboratory of Quality Research in Chinese Medicine, Institute of Chinese Medical Sciences, University of Macau, Avenida da Universidade, Taipa, Macao SAR, 999078 China; 2 MoE Frontiers Science Center for Precision Oncology, University of Macau, Macao SAR, 999078 China; 3 College of Pharmaceutical Science, Zhejiang University of Technology, Hangzhou, 310014 China; 4 Guangxi Key Laboratory of Bioactive Molecules Research and Evaluation & Pharmaceutical College, Guangxi Medical University, Nanning, 530021 China; 5 National-Local Joint Engineering Laboratory of Druggability and New Drug Evaluation, Guangdong Key Laboratory of Chiral Molecule and Drug Discovery, School of Pharmaceutical Sciences, Sun Yat-Sen University, Guangzhou, 510006 China; 6 Department of Pharmaceutical Sciences, Faculty of Health Sciences, University of Macau, Macao SAR, 999078 China

**Keywords:** Lycorine, Lycorine hydrochloride, Anticancer mechanism, Target, Pharmacokinetics

## Abstract

**Background:**

Natural products have a long history in drug discovery. Lycorine is an alkaloid derived from *Amaryllidaceae* plants, demonstrating significant pharmacological potential. Lycorine and its hydrochloride salt, lycorine hydrochloride, have shown outstanding anticancer effects both in vitro and in vivo.

**Purpose:**

This review aims to comprehensively summarize recent research advancements regarding the anticancer potential of lycorine and lycorine hydrochloride. It intends to elucidate current research limitations, optimization strategies, and future research directions to guide clinical translation.

**Methods:**

Various databases, *e.g.*, Web of Science, PubMed, and Chinese National Knowledge Infrastructure, are systematically searched for relevant articles using keywords such as lycorine, cancer, pharmacokinetics, and toxicity. The retrieved literature is then categorized and summarized to provide an overview of the research advancements in the anticancer potential of lycorine and lycorine hydrochloride.

**Results:**

Lycorine and lycorine hydrochloride demonstrate significant anticancer activities against various types of cancer both in vitro and in vivo, employing diverse mechanisms such as inducing cell cycle arrest, triggering cellular senescence, regulating programmed cell death, inhibiting angiogenesis, suppressing metastasis, and modulating immune system. Furthermore, pharmacokinetic profiles and toxicity data are summarized. Additionally, this review discusses the druggability, limitations, optimization strategies, and target identification of lycorine, offering insights for future preclinical studies.

**Conclusion:**

The anticancer effects and safety profile of lycorine and lycorine hydrochloride suggest promising potential for clinical applications. Further research on their in-depth mechanisms and optimization strategies targeting their limitations will enhance the understanding and druggability of lycorine and lycorine hydrochloride.

**Graphical Abstract:**

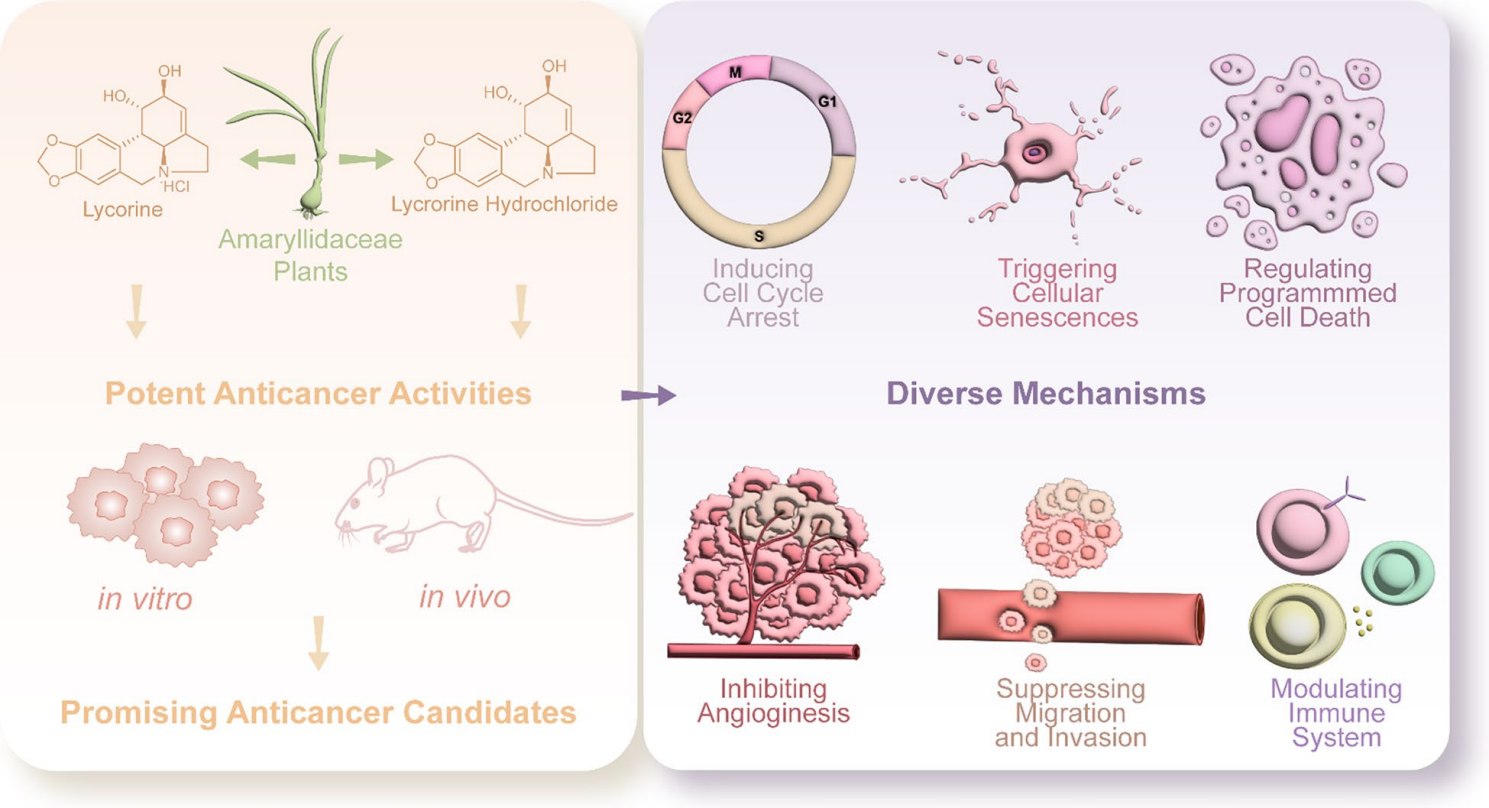

## Introduction

Natural products have made significant contributions to drug discovery for millennia, owing to their rich chemical diversity and pharmacological activities [1]. These compounds represent a vital resource for treating a wide range of diseases [2]. As an illustration, metformin, derived from *Galega officinalis* and modified, has been utilized since the seventeenth century for diabetes treatment and demonstrated additional pharmacological benefits beyond its initial application [3]. Similarly, artemisinin-derived artesunate is a frontline treatment for severe malaria, and digoxin from *Digitalis lanata* is extensively employed for managing cardiac conditions such as heart failure, atrial fibrillation, and so on [4–6]. The historical use and observed benefits of natural-derived drugs underscore their significance in drug development.

Cancer remains a major global health challenge, necessitating the continual search for novel therapeutic agents [7]. In the realm of anticancer drug discovery, natural products and their derivatives have been particularly influential, providing a substantial number of widely used anticancer agents [8–10]. For example, paclitaxel (PTX), derived from *Taxus brevifolia*, effectively treats cancer by promoting tubulin polymerization and disrupting mitotic progression [11]. Camptothecin, derived from *Camptotheca acuminata*, inhibits DNA synthesis via topoisomerase I, with its derivative Irinotecan achieving significant clinical success since its U.S. Food and Drug Administration (FDA) approval in 1994 [12]. Additionally, arsenic trioxide used to treat acute promyelocytic leukemia by degrading PML-RARα fusion protein, has shown 10-year survival rates of over 80% when combined with all-trans retinoic acid [13]. The multitude of natural products utilized in cancer treatment emphasize their indispensable role in the quest for more effective and less toxic anticancer drugs.

**Fig. 1:**
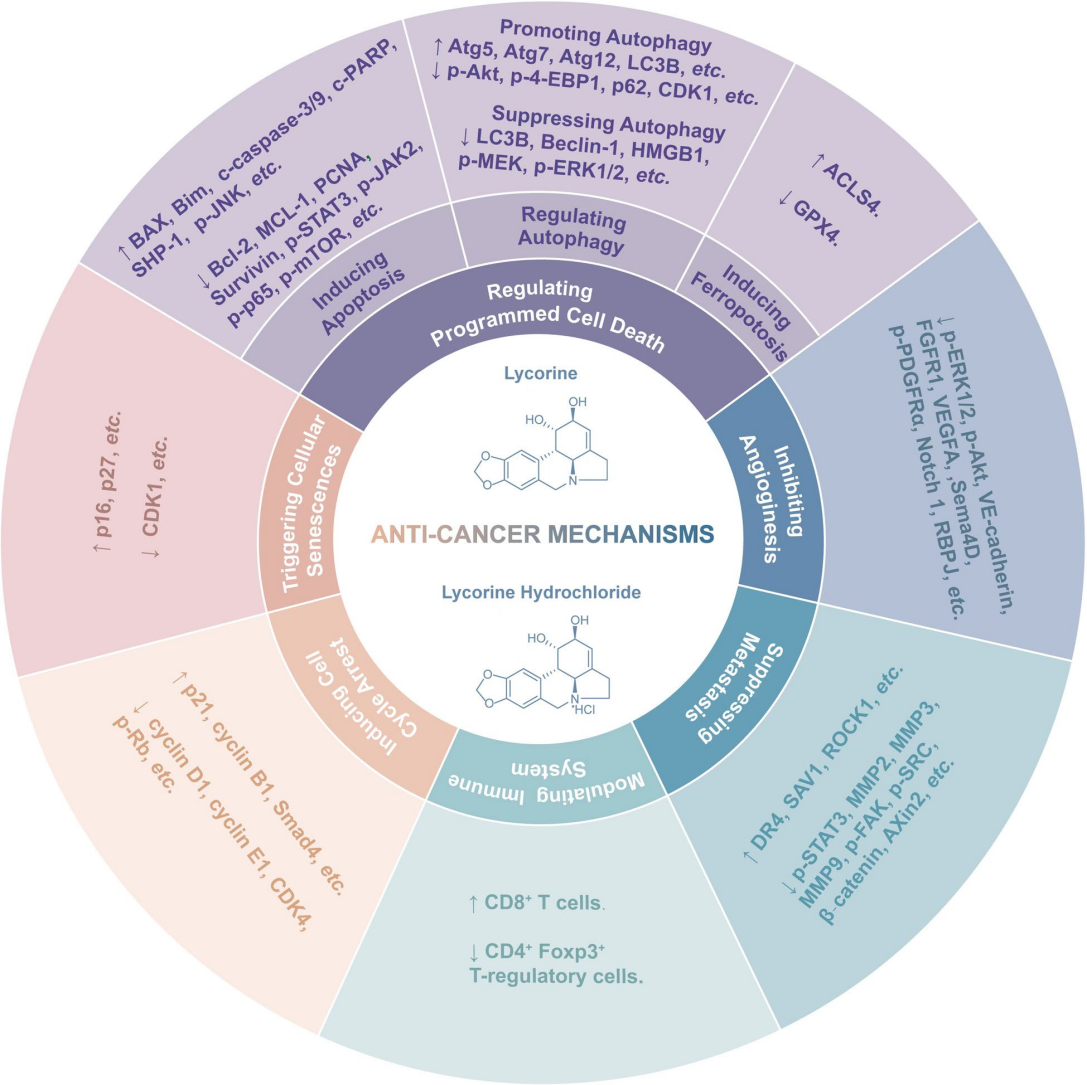
Chemical structures and anti-cancer mechanisms of lycorine and lycorine hydrochloride. The two chemical structures represent lycorine and lycorine hydrochloride, respectively. Anti-cancer mechanisms of lycorine and lycorine hydrochloride include inducing cell cycle arrest, triggering cellular senescence, regulating programmed cell death, suppressing migration and invasion, inhibiting angiogenesis, and modulating immune system. Different molecules regulated by lycorine and lycorine hydrochloride in these processes are classified and shown

In the expansive bounty of nature, *Amaryllidaceae* plants are widely distributed in tropical and subtropical regions worldwide [14]. Their use in traditional medicine dates back thousands of years [15, 16]. Among the alkaloids derived from these plants are lycorine, galanthamine, lycoramine, and others, which exhibit a wide range of pharmacological activities [17–19]. Galanthamine, for instance, recognized as an acetylcholinesterase inhibitor, obtained approval from the FDA for Alzheimer's disease treatment in 2001, highlighting the pharmacological potential and safety of this plant family. Lycorine is another prominent alkaloid sourced from various *Amaryllidaceae* plants including *Lycoris radiata* (a traditional Chinese medicinal herb) [20], *Leucojum aestivum* [21]*, Ungernia sewertzowii* [22], etc*.* Its structural identification was first completed in 1956 [23]. Lycorine has gained considerable attention for its diverse pharmacological activities including antiviral effects[24], antibacterial effects [25], antifungal effects [26], anti-inflammatory effects [24], fibrosis inhibition [27], Parkinson's disease treatment [28], organ protection [29], metabolism regulation [18], analgesic effect [30], and so on. Particularly noteworthy is lycorine ‘s anticancer activity, extensively studied in both cellular and animal models through various mechanisms [25, 31]. Given lycorine’s relatively poor solubility [32], lycorine hydrochloride, the hydrochloride salt form of lycorine, sharing a similar structure and displaying similar pharmacological effects, may be used as an alternative in many studies due to its better water solubility. The chemical structures of lycorine and lycorine hydrochloride are shown in Fig. 1.

The low toxicity of lycorine and lycorine hydrochloride along with their potent anticancer activities, positions them as promising candidates for anticancer drug development. Despite past reviews summarizing the anticancer effects of lycorine [25, 31], recent advancements have expanded our understanding, revealing novel insights into their mechanisms, including the identification of direct molecular targets and strategies to enhance druggability, areas previously underexplored [33–37]. This review comprehensively concludes the in vitro and in vivo anticancer activities, molecular mechanisms, pharmacokinetics, and toxicity of lycorine and lycorine hydrochloride, while also addressing current limitations and potential clinical applications, offering insights for future research endeavors.

## Methods

This review article summarized the literature published before July 2024. Various databases, including Web of Science, PubMed, and Chinese National Knowledge Infrastructure, were systematically searched for relevant articles using keywords such as lycorine, lycorine hydrochloride, cancer, target, pharmacokinetics, toxicity, and derivative. The literature was screened and irrelevant articles were excluded. The quality of the remaining articles was assessed, and those with obvious errors, unclear information, or duplicate content were removed [38]. Subsequently, the retrieved literature was then categorized and summarized to provide an overview of the research advancements in the anticancer potential of lycorine and lycorine hydrochloride. Moreover, the discussion proposes future research directions for lycorine and lycorine hydrochloride.

## Anticancer activities of lycorine and lycorine hydrochloride

### Single treatment

Lycorine and lycorine hydrochloride have demonstrated significant anticancer effects both in vitro and in vivo. They exhibit proliferation inhibitory effects on various types of cancer cells including bladder cancer [39, 40], breast cancer [17, 41–43], B-cell acute lymphoblastic leukemia [44], cervical cancer [17, 45], colorectal cancer [17, 46], gastric cancer [17, 47], glioma [48], liver cancer [49–52], leukemia [17, 53], glioblastoma multiforme [54], lung cancer [17, 55–57], melanoma [58, 59], myeloma [60, 61], oral cancer [62], osteosarcoma [17, 63–65], ovarian cancer [17, 66], pancreatic cancer [17, 67], and so on. IC_50_ values range from 0.8 μM to 50 μM within an administration time from 12 to 72 h, as indicated in Table 1. The sensitivities to lycorine and lycorine hydrochloride differed across various types of cancer cells. Typically, lycorine and lycorine hydrochloride inhibited proliferation at low concentrations with IC_50_ values less than 5 μM with administration of 48 h or more in most cancer cells, such as colorectal cancer, leukemia, ovarian cancer, pancreatic cancer, and so on. The proliferation effects are shown in time- and dose-dependent manners. However, there are discrepancies in IC_50_ values reported for the same cell types under similar treatment conditions across different studies. These variations in sensitivity among cancer cells may be attributed to differences in cell density, serum concentration used, detection methods, etc. Nonetheless, it is noteworthy that lycorine and lycorine hydrochloride consistently demonstrate effective anticancer properties.

**Table 1:** The anti-proliferation activities of lycorine and lycorine hydrochloride in vitro

Cancer type	Cell line	Time (h)	Method	IC_50_ (μM)	Reference
Bladder cancer	T24	72	CCK-8	~ 1	[39]
	5673	72	CCK-8	~ 1	
Bladder cancer	T24	48	MTT	7.5	[40]
Breast cancer	BT-549	48	CCK-8	1.1	[17]
	MCF-7	48	CCK-8	0.8	
	MDA-MB-231	48	CCK-8	1.4	
Breast cancer	MDA-MB-231	48	SRB	0.9	[41]
	MDA-MB-468	48	SRB	1.4	
	MCF-7	48	SRB	0.7	
Breast cancer	MCF-7	48	SRB	0.5–2	[42]
	T47D	48	SRB	2–5	
	MDA-MB-231	48	SRB	5–10	
	4T1	48	SRB	2–5	
Breast cancer	MDA-MB-231	48	CCK-8	40–60	[43]
	MCF-7	48	CCK-8	40–60	
Breast cancer	MDA-MB-231	24	MTT	1.9	[36]
	MCF-7	24	MTT	7.8	
B-cell acute lymphoblastic leukemia	REH	72	MTS	1.6	[44]
	NALM-6	72	MTS	1.9	
Cervical cancer	HeLa	48	CCK-8	0.9	[17]
Cervical cancer	HT-3	24	MTT	40.0	[45]
Colorectal cancer	HCT116	72	MTT	1.4	[73]
	LoVo	72	MTT	3.8	
	SW480	72	MTT	1.3	
Colorectal cancer	COLO-201	48	CCK-8	0.8	[17]
	HT-29	48	CCK-8	0.8	
	SW-480	48	CCK-8	0.9	
Gastric cancer	AGS	48	CCK-8	0.8	[17]
Glioma	C6	48	MTT	2.9	[48]
Liver cancer	HepG2	48	MTT	2.6	[52]
	Huh-7	48	MTT	2.0	
Liver cancer	HepG2	48	MTT	2–10	[50]
Liver cancer	HepG2	48	MTT	34.1	[51]
	SMMC-7721	48	MTT	36.6	
	Huh-7	48	MTT	38.3	
Leukemia	HL-60	24	MTT	~ 1	[53]
Leukemia	HL-60	48	CCK-8	1.3	[17]
	Jurkat	48	CCK-8	1.3	
	MOLT-4	48	CCK-8	1.0	
Glioblastoma multiforme	U251	48	SRB	10–20	[54]
	U87	48	SRB	10–20	
	A172	48	SRB	10–20	
	LN299	48	SRB	10–20	
Lung cancer	SPC-A-1	72	CTG	4.8	[55]
	A549	72	CTG	4.0	
Lung cancer	A549	24	MTT	10.8	[56]
	H460	24	MTT	12.4	
Lung cancer	A549	24	CCK-8	5–10	[57]
	H1299	24	CCK-8	10–20	
Lung cancer	A549	48	CCK-8	1.0	[17]
	H1299	48	CCK-8	0.9	
Melanoma	A375	48	MTT	~ 20	[58]
	MV3	48	MTT	~ 20	
Melanoma	A375	72	CCK-8	~ 50	[59]
Myeloma	ANBL6	24	CCK-8	2.5–5	[61]
	MM.1S	24	CCK-8	~ 10	
Oral cancer	HSC-3	48	CCK-8	6.2	[62]
	HSC-4	24	CCK-8	48.6	
	UM1	24	CCK-8	35.3	
	UM2	24	CCK-8	28.0	
Osteosarcoma	Saos-2	48	CCK-8	1.2	[17]
Osteosarcoma	HOS	24	CCK-8	3.0	[63]
	MG63	24	CCK-8	2.5	
	U2OS	24	CCK-8	1.4	
	SJSA-1	24	CCK-8	1.9	
Osteosarcoma	143B	48	MTT	3.2	[64]
	MG63	48	MTT	5.5	
	Saos-2	48	MTT	2.9	
	U2OS	48	MTT	3.2	
Osteosarcoma	MNNG/HOS	48	CCK-8	1–2	[65]
	U2OS	48	CCK-8	~ 2,	
	MG63	48	CCK-8	~ 2	
	Saos-2	48	CCK-8	~ 2	
Ovarian cancer	A2780	48	CCK-8	1.2	[17]
Ovarian cancer	Hey1B	48	AlamarBlue	1.2	[66]
Pancreatic cancer	Panc-1	72	CTG	1.0	[67]
	Patu8988	72	CTG	1.5	
Pancreatic cancer	PANC-1	48	CCK-8	0.7	[17]

The therapeutic efficacy on mouse models further demonstrated the remarkable anticancer potential of lycorine and lycorine hydrochloride. Lycorine and lycorine hydrochloride were administered in vivo through various routes mainly by oral gavage (*i.g.*), intraperitoneal injection (*i.p.)*, or intravenous injection (*i.v.*). Administering 5–30 mg/kg lycorine and lycorine hydrochloride significantly inhibited tumor growth in the xenograft mouse model, mostly conducted using nude mice and BALB/c mice. Notably, the administration of 20 mg/kg/day of lycorine for 40 days via *i.p.* injection resulted in almost complete tumor suppression in luciferase-expressing U251-bearing nude BALB/c mice, suggesting the potent anticancer efficacy of lycorine [54]. In addition to tumor growth inhibition, lycorine and lycorine hydrochloride significantly suppressed metastasis. For example, *i.p.* injection of 5 mg/kg lycorine for 30 days suppressed lung metastasis by about 80% in the tail vein MDA-MB-231-Luc injection model, with comparable efficacy to paclitaxel but with less weight loss and reduced toxicity [42]. Similarly, *i.p.* injection of 10 mg/kg lycorine hydrochloride for 28 days significantly suppressed over 40% of lung metastasis in C8161 tumor-bearing mice with [68]. Furthermore, in SPC-A-1-bearing nude mice, *i.p* injection of 10 mg/kg lycorine remarkably inhibited metastasis in the A549 metastasis model [55]. Additionally, *i.p.* injection of 30 mg/kg lycorine hydrochloride inhibited tumor growth and metastasis in both the A375 xenograft and metastasis model, with no significant differences in body weight observed [58]. The in vivo anticancer activities are summarized in Table 2. Based on the aforementioned results, lycorine and lycorine hydrochloride exhibit highly effective tumor-suppressive effects with minimal toxicity in animal models. At the comparable dosage, they not only effectively inhibit tumor growth but also suppress metastasis, offering promising potential in restraining cancer progression.

**Table 2:** The anticancer activities of lycorine and lycorine hydrochloride in vivo

Cancer type	Animal model	Cell line	Mode of administration	Effects	Reference
Breast cancer	Nude mice^a^	MDA-MB-231	5, 10 mg/kg/day for 30 days, *i.p.*	Tumor growth inhibition rates were about 60%, 70%	[42]
	Nude mice	4T1	5, 10 mg/kg/day for 21 days, *i.p.*	Tumor growth inhibition rates were about 40%, 50%	
	Nude mice	MDA-MB-231	5 mg/kg/day for 21 days, i.p.	Lung metastasis inhibition rate was about 80%	
Breast cancer	BALB/c mice	4T1/Luc	5, 15 mg/kg/day for 28 days, *i.p.*	Tumor growth inhibition rates were about 30%, 50%	[36]
Breast cancer	Nude mice	MDA-MB-231	10 mg/kg/day for 18 days, *i.p.*	Tumor growth inhibition rates were about 75%	[43]
Bladder cancer	Nude mice	T24	5, 10 mg/kg/day for 14 days, *i.p.*	Tumor growth inhibition rates were 63.50%,74.21%	[40]
B-cell acute lymphoblastic leukemia	NCG mice	NALM-6	10, 20 mg/kg/bid for 21 days, *i.p.*	Tumor growth was significantly inhibited	[44]
Colorectal cancer	BALB/c mice	CT26	20 mg/kg/day for 14 days, *i.p.*	Tumor growth inhibition rate was about 65%	[33]
Colorectal cancer	BALB/c mice	CT26	10, 40 mg/kg/day for 14 days,* i.v.*	Suppressed lung metastasis to 60.99% and 54.14%	[46]
Colorectal cancer	BALB/c nude mice	SW480	20 mg/kg/day for 33 days, *i.p.*	Tumor growth inhibition rate was about 65%	[102]
Gastric cancer	Nude mice	Patient -derived tissue	Lycorine hydrochloride (30 mg/kg) + HA14–1 (2.5 mg/kg) for 13 days, N/A	Tumor growth inhibition rate was about 70%	[47]
Glioblastoma	BALB/c nude mice	U251-luc	10, 20 mg/kg/day for 40 days, *i.p.*	Tumor growth inhibition rates were 87.20%, 97.98%	[54]
	BALB/c nude mice	Patient-derived glioblastoma multiforme cell	10, 20 mg/kg/day for 14 days, *i.p.*	Tumor growth inhibition rates were 54.72%, 75.14%	
Liver cancer	BALB/c nude mice	Huh7	Lycorine 10 mg/kg/day for 30 days, *i.g.*	Tumor growth inhibition rate was about 25%	[37]
			Sorafenib 30 mg/ kg/day for 30 days, *i.g…*	Tumor growth inhibition rate was about 60%	
			Lycorine 10 mg/kg/day + Sorafenib 30 mg/ kg/day for 30 days, *i.g.*	Tumor growth inhibition rate was over 90%	
Liver cancer	C57BL/6 mice	H22	10 mg/kg every 2 days for 18 days, *i.p.*	Tumor growth inhibition rate was about 60%	[78]
Liver cancer	BALB/c nude mice	HepG2	10,20 mg/kg/day for 33 days, *i.p.*	Tumor growth inhibition rates were about 50%, 33%	[51]
Leukemia	BALB/c SCID mice	HL-60	5, 10 mg/kg/day on day 2 to 6 and day 14 to 18, *i.p.*	Decrease the percentages of immature granular leukocytes and monocytes among the peripheral blood cells, prolong survival period	[137]
Lung cancer	BALB/c nude mice	A549/Luc	2.5,5 mg/kg every 2 days for 28 days, *i.p.*	Tumor growth inhibition rates were 71.04%, 82.27%	[56]
Lung cancer	Nude mice	SPC-A-1	10 mg/kg/day for 28 days, *i.p…*	Tumor growth inhibition rate was over 75%	[55]
		A549	10 mg/kg/day for 34 days, *i.p.*	Suppressed lung metastasis significantly	
Melanoma	NOD/SCID mice	A375	30 mg/kg/day for 25 days, *i.p.*	Tumor growth inhibition rate was about 50%	[58]
			30 mg/kg/day for 40 days, *i.p.*	Lung metastasis inhibition rate was about 75%	
Melanoma	Nude mice	C8161	10 mg/kg/day for 28 days, *i.p.*	Lung metastasis inhibition rate was about 40%	[68]
Myeloma	NOD/SCID mice	MM.1S	Lycorine 5 mg/kg on day 1,2,3,5,6,7,9,10,11, *i.p.*	Tumor growth inhibition rate was 48.06%	[61]
			BTZ (1 mg/kg/ every 4 days for 11 days), *i.p.*	Tumor growth inhibition rate was 41.41%	
			Lycorine 1 mg/kg on day 2, 3, 6, 7, 10, 11 + BTZ 0.25 mg/kg on day 1, 5, 8, *i.p.*	Tumor growth inhibition rate was 73.53%	
Ovarian cancer	BALB/C nude mice	Hey1B	15 mg/kg/day for 24 days, *i.p.*	Tumor growth inhibition rate was 61.64%	[66]
Pancreatic cancer	C57BL/6 mice	PAN02	5,10 mg/kg for 20 days, *i.p.*	Tumor growth inhibition rates were about 75%, 85%	[34]
Prostate cancer	C57/BL mice	RM-1	5, 10 mg/kg/day for 14 days, *i.g.*	Tumor growth inhibition rates were about 40%, 50%	[72]

^a^Some studies refer to “Nude mice” without specifying the strain, while others specifically mention “BALB/c nude mice.” Both types of references are included in the table to provide a more comprehensive overview

### Combined treatment

In addition to single treatment, lycorine and lycorine hydrochloride have demonstrated enhanced effects of anticancer activities in combination therapy as evidenced by both in vivo and in vitro experiments. For instance, in the hepatocellular carcinoma xenograft BALB/c nude mice model, 10 mg/kg lycorine with 30 mg/kg sorafenib via *i.g.* administration significantly inhibited tumor growth compared to single treatments [37]. Lycorine combined with temozolomide, a first-line agent for the treatment of glioma, inhibited the proliferation of glioma cells [48]. Moreover, combining 30 mg/kg lycorine hydrochloride with Bcl-2 inhibitor HA14-1 demonstrated increased tumor inhibition compared to HA14-1 alone in a gastric patient-derived xenograft model [47]. Similarly, in MM.1S xenograft NOD/SCID mice model, the tumor inhibition rate in the combined treatment group was twice higher than in the single treatment group of lycorine or bortezomib (BTZ) alone [61]. Furthermore, chronic lymphocytic leukemia (CLL) cells keep proliferation and evade apoptotic signals by binding to CD40 ligands (CD40L) expressing T cells in lymph nodes [69]. A combination of bezafibrate, medroxyprogesterone acetate, and lycorine significantly induced apoptosis in CLL cells exposed to CD40L, an effect not achieved by bezafibrate or medroxyprogesterone acetate alone [69]. Beyond merely enhancing the efficacy of combination therapies with other drugs, lycorine and lycorine hydrochloride playing crucial roles in mitigating drug resistance. For example, drug resistance frequently leads to tumor recurrence in breast cancer patients despite the use of tamoxifen, an estrogen receptor antagonist employed in treatment. In tamoxifen-resistant MCF-7 and T47D cells, lycorine combined with 5 μM tamoxifen increased apoptosis rate and inhibited cell viability compared to lycorine or tamoxifen treatment alone, resulting in alleviating drug resistance [35]. In addition, tyrosine kinase inhibitor sorafenib is widely used in advanced hepatocellular carcinoma treatment, and lycorine effectively reversed the acquired resistance of sorafenib both in vitro and in vivo. Additionally, in Renca-Luc xenograft C57BL/6 mice, a combination of 5 mg/kg lycorine hydrochloride and cytotoxic T-lymphocyte associated protein 4 (CTLA-4) antibody significantly suppresses metastasis compared to single treatments [70]. All these data underscore the potential of lycorine and lycorine hydrochloride to enhance the efficacy of various chemotherapy drugs, improve immunotherapy outcomes, and overcome drug resistance. The in vivo combination effects of lycorine and lycorine hydrochloride are also shown in Table 2.

## Anticancer mechanisms of lycorine and lycorine hydrochloride

The potential mechanisms underlying anticancer activities of lycorine and lycorine hydrochloride, which are all contribute to their potent anticancer activities, including inducing cell cycle arrest, triggering cellular senescence, regulating programmed cell death, suppressing migration and invasion, inhibiting angiogenesis, as well as modulating immune system (Fig. 1).

### Inducing cell cycle arrest

Continuous cell cycle progression is a hallmark of cancer cells presenting an opportunity for therapeutic intervention by inducing cell cycle arrest to impede aberrant division and cancer cell proliferation [71]. Lycorine and lycorine hydrochloride induced cell cycle arrests in different phases depending on the cancer cell types. Lycorine and lycorine hydrochloride with a concentration range of 2.5–40 μM induced cell cycle arrest in the G2/M phase in various cancer cell lines including breast cancer cells MCF-7 and MDA-MB-231 [36], prostate cancer cells PC3 and DU145 [72], colorectal cancer cells HCT116 and LoVo [73], leukemia cell HL60 [53], ovarian cancer cell Hey1B [66], pancreatic cancer cells PANC-1 and BxPC-3 [34], and hepatocellular carcinoma cells HepG2 [50], and so on. For example, treatment with 3.3 μM lycorine hydrochloride increased cells in the G2/M phase from 13.2% to 26.8% in Hey1B [66]. In addition, 2–20 μM lycorine and lycorine hydrochloride induced cell cycle arrest in G0/G1 phase in oral squamous cell carcinoma HSC‑3 cell [62], leukemia cells K562 [74], osteosarcoma cells HOS and U2OS [65]. While in melanoma cells MV3 and A375 [58] and gastric cancer cells MKN-45 and SGC7901 [47], 48 h treatment of 20 μM lycorine hydrochloride induced cell cycle arrest in the S phase, potentially resulting in cellular dysfunction such as DNA damage, and subsequently initiating apoptosis. The diverse effects of lycorine and lycorine hydrochloride on cell cycle arrest at different stages may be attributed to their binding to multiple targets in various tumor cells. Additionally, variations in drug administration durations and doses, as well as inherent differences in cell cycle processes among cells, may contribute to distinct cell cycle arrests.

Cell cycle progression is regulated by some key factors. Cyclin-dependent kinases (CDKs) form active complexes with cyclins, expressed at specific cell cycle stages, to phosphorylate the retinoblastoma protein (Rb) and drive the cell cycle forward. When cells experience stress or DNA damage, p53 is activated leading to cell cycle arrest for DNA repair through the transcriptional activation of p21 [71]. In cancer cells arrested by lycorine and lycorine hydrochloride, alterations in the protein levels of p53, p21, cyclins, and CDKs complexes at specific cell cycle phases were observed. Specifically, lycorine was found to increase cyclin B1 and Smad4 but decrease cyclin D1 and cyclin E1 in HCT116 and LoVo cells [73]. Moreover, lycorine functioned as a histone deacetylase inhibitor, increasing p53 and p21, reducing cyclin D1, CDK4, and phosphorylated p-Rb, which further induced cell cycle arrest in K562 chronic myelocytic leukemia cells [74]. Cell cycle arrest induced by lycorine and lycorine hydrochloride finally inhibited cancer cell proliferation.

### Triggering cellular senescence

Cellular senescence refers to cells entering into permanent and irreversible cell cycle arrest after sustaining stresses [75]. Characterized by its inhibition effect on tumorigenesis, cellular senescence is considered to be a potential cancer therapy strategy [76, 77]. In hepatocellular carcinoma cells Hep3B and HuH-7, increased SA-β-gal activity by lycorine was evidence of cellular senescence. Lycorine induced cellular senescence and effectively inhibited tumor growth by increasing p16 and p27 and decreasing CDK 1 both in vitro and in vivo [78]. In these two cell lines, senescence occurred accompanied by autophagy but with no apoptosis detected, although lycorine-induced apoptotic cells were detected in other hepatocellular carcinoma cells (HepG2 and SMMC-7721) [51]. However, despite advancements in detecting tumor senescence, there is a dearth of research on whether lycorine and lycorine hydrochloride have similar effects in different types of cancer cells, requiring further investigation. Additionally, it has been found that a class of pro-inflammatory SASP factors secreted by senescent tumor cells created an immunosuppressive tumor microenvironment (TME), leading to tumor immune evasion and promoting metastasis after treatment [79]. The potential involvement of lycorine and lycorine hydrochloride in the clearance of senescent cells within the TME remains unclear and necessitates deeper investigation. Combining lycorine and lycorine hydrochloride with senolytics (drugs designed to clear senescent cells) may be explored as a more effective strategy to suppress tumor growth.

### Initiating programmed cell death

#### Inducing apoptosis

Apoptosis is a form of programmed cell death that has been an effective strategy for targeting cancer cells [80]. Administration of lycorine and lycorine hydrochloride at concentrations ranging from 2 to 50 μM induced varying levels of apoptosis in a variety of cancer cell lines, including acute myeloid leukemia [81], breast cancer [35, 42, 43], bladder cancer [40], colorectal cancer [33, 46], gastric cancer [47], glioma [48], liver cancer [51], lung cancer [57], multiple myeloma [60], oral squamous cell carcinoma [62], osteosarcoma [65], pancreatic cancer [67], prostate cancer [72]. For example, in gastric cancer cells MKN-45 and SGC-7901, exposure to the concentration of IC_50_ (20 μM) of lycorine hydrochloride resulted in apoptosis rates exceeding 40% [47], suggesting that apoptosis may be responsible for tumor suppression.

The apoptotic mechanisms induced by lycorine and lycorine hydrochloride vary depending on the specific cancer type with both intrinsic and extrinsic apoptotic pathways being observed. Lycorine and lycorine hydrochloride can trigger intrinsic apoptosis by modulating the permeability of mitochondrial membranes through the regulation of the Bcl-2 protein family [60, 62, 65, 72]. Subsequent activation of the caspase cascade led to upregulation of cleaved caspase-3 (c-cas 3), cleaved caspase-9 (c-cas 9), and cleaved PARP (c-PARP), ultimately driving apoptosis [42, 43, 47, 51, 72]. In gastric cancer cells, lycorine hydrochloride induced ubiquitin-mediated degradation of MCL1 by increasing ubiquitin E3 ligase FBXW7 [47]. In acute myeloid leukemia cells, lycorine bound to fatty acid binding proteins 5 (FABP5), reducing triglyceride production and consequently increasing apoptotic rates [81]. Moreover, lycorine and lycorine hydrochloride triggered the overproduction of reactive oxygen species (ROS), leading to apoptosis in C6 and HSC‑3 cells [48, 62]. Lycorine also induced apoptosis via downregulating the expression of HOXD antisense growth-associated lncRNA (HAGLR), which was reported as an oncogene in breast cancer cells [35]. In contrast, lycorine hydrochloride induced extrinsic apoptosis in colorectal cancer cells and increased the mRNA expression of *DR4, DR5, GADD45A*, and *TRAF2*, while also reducing protein levels of PCNA, PARP-1, and Survivin, thereby inducing death receptor-mediated extrinsic apoptosis [46].

Lycorine and lycorine hydrochloride induced apoptosis in cancer cells by regulating multiple intracellular pathways including NF-κB [48, 72], JAK2/STAT3 [65], and JNK [62] pathways, which are crucial for cancer cell survival and growth. Lycorine upregulated the expression of SH2 domain-containing phosphatase 1 (SHP-1), leading to the inhibition of p-STAT3 [65]. Lycorine inhibited IkBα and increased p-p65 in C6 cells [48]. Furthermore, lycorine hydrochloride activated the JNK signaling pathway by increasing protein levels of p‑MKK4 and p‑JNK in HSC-3 cells, contributing to apoptosis induction [62]. Other key pathways mediating apoptosis were likely regulated by lycorine and lycorine hydrochloride as well. Evidence from RNA sequencing analysis showed that several signaling pathways, such as MAPK and PI3K-Akt, were significantly upregulated [46], but further experimental validation was lacking. The most frequently observed mode of cell death following lycorine and lycorine hydrochloride administration is apoptosis, which is believed to play a crucial role in tumor suppression across various types of cancers. By inducing apoptosis in cancer cells, lycorine and lycorine hydrochloride may effectively inhibit tumor growth and progression, underscoring their potential as promising therapeutic agents for cancer treatment and offering a targeted approach to combat malignant cell proliferation and enhance overall therapeutic outcomes.

#### Regulating autophagy

Autophagy is a regulatory mechanism that maintains protein homeostasis and organelle integrity and functionality within cells. In cancer therapy, autophagy plays a complex and dual role, exerting both pro-survival and pro-death effects on cells [82]. Many natural products derived from traditional Chinese medical herbs have been shown to modulate autophagy, like platycodin D, curcumin, paclitaxel, resveratrol, etc. [83, 84]. The influence of lycorine on autophagy is context-dependent, with its effects varying across different cell types and tumor genetic backgrounds. In tumor cells where lycorine inhibits proliferation, both autophagy inhibition and promotion can occur. For instance, in hepatocellular carcinoma cancer cells, 2.5–40 μM lycorine promoted autophagy and exhibit cell proliferation inhibition [51, 78]. In HepG2 and SMMC-7721 cells, lycorine inhibited p-Akt and p-4-EBP1, leading to increased autophagy-related proteins (Atg5, Atg7, Atg12, and LC3B) and decreased protein level of p62, thereby inducing autophagy [51]. Conversely, 5–10 μM lycorine inhibited autophagy in multiple myeloma cells and suppressed tumor growth [61]. Lycorine reduced LC3-II and Beclin-1 protein levels in multiple myeloma cells by promoting HMGB1 degradation and suppressing the MEK-ERK pathway and inhibiting autophagy, further inhibiting proliferation [61]. Additionally, some instances of lycorine-induced autophagy are accompanied by apoptosis [51, 85], suggesting that autophagy alone might not be sufficient to explain the inhibitory effects on cell proliferation. It is possible that autophagy plays a limited role in the inhibition of cell proliferation, with its interaction with apoptosis potentially collectively influencing cancer cell fate. Furthermore, the role of autophagy in cancer can also vary depending on the stage of tumor progression [82]. Whether lycorine and lycorine hydrochloride promote or inhibit autophagy are not able to reflect their efficacy in cancer treatment. Therefore, determining whether lycorine promotes or inhibits autophagy may not be the focal point.

#### Inducing ferroptosis

Ferroptosis, a distinct form of programmed cell death characterized by lipid peroxidation, iron overload, and ROS, has been identified as a mechanism capable of inhibiting tumor growth [86]. Acyl-CoA synthetase long-chain family member 4 (ACSL4) is required for lipid peroxidation by activating ROS generation in the progress of ferroptosis [87]. Lycorine was found to induce ferroptosis and suppress proliferation in renal cell carcinoma cells 786‑O, A498 and Caki‑1 by downregulating GPX4 and upregulating ACSL4 expression [88]. Ferroptosis, a newly defined form of programmed cell death in 2012 [89], may be overlooked in earlier studies on lycorine and lycorine hydrochloride. Increasing studies suggest that lycorine and lycorine hydrochloride can regulate lipid metabolism [34, 37, 81], and ferroptosis is closely associated with the oxidation of various lipids [89]. Therefore, it is plausible that lycorine and lycorine hydrochloride may influence ferroptosis through the regulation of lipid metabolism in cancer cells, presenting a promising direction for further investigation to explore the potential role of lycorine and lycorine hydrochloride in regulating ferroptosis and its implications for cancer therapy.

### Inhibiting angiogenesis

Aberrant angiogenesis is one of the indicators of tumor tissue and promotes tumor immune escape [90]. Depriving oxygen and nutrients to inhibit angiogenesis represents a potential therapeutic approach to suppress tumor growth [91]. Furthermore, angiogenesis plays a pivotal role in the early metastasis of tumors, emphasizing its significance as a therapeutic target to mitigate cancer metastasis [92]. Lycorine showed significant inhibitory effects on the proliferation and migration of primary umbilical vein endothelial cells (HUVECs) in a concentration- and time-dependent manner, with IC_50_ values of 9.34 µM at 24 h and 4.93 µM at 48 h. The ex vivo Chick chorioallantoic membrane (CAM) model confirmed the anti-angiogenic ability of lycorine by significantly inhibiting neovascularization compared to the control group [93]. Moreover, lycorine hydrochloride was found to suppress the formation of blood vessels in A549-, SPC-A-1- and Hey1B- bearing nude mice using Matrigel plug assay [55, 66]. It was also observed that lycorine hydrochloride inhibited angiogenesis in both C8161 melanoma cells and C8161 tumor-bearing nude mice [94].

Lycorine hydrochloride treatment resulted in the inhibition of many pro-angiogenesis genes, including *VE-cadherin, FGFR1, VEGFA, and Sema4D* [66, 67, 94]. Lycorine and lycorine hydrochloride inhibited angiogenesis through the suppression of EGFR-Akt signaling pathways in ovarian cancer cell [66] and PDGFRα-Akt signaling pathways in CAM model [93]. In pancreatic cancer, lycorine hydrochloride inhibited angiogenesis and tumor growth by promoting ubiquitin-mediated degradation of Notch1 and its downstream effector, recombination signal binding protein for immunoglobulin kappa J region (RBPJ) [67]. Above all, lycorine and lycorine hydrochloride demonstrate the ability to modulate angiogenic genes, effectively inhibiting vascular mimicry and significantly suppressing tumor growth. Their inhibitory effects on angiogenesis may further contribute to the suppression of metastasis.

### Suppressing migration and invasion

Metastasis, the process by which cells from a primary tumor spread to distant sites, accounts for the majority of cancer-related deaths [95]. The migration and invasion of tumor cells are necessary processes of metastasis [96]. Methods such as wound healing and transwell assays were employed to confirm that lycorine and lycorine hydrochloride, at concentrations ranging from 1 to 40 μM, exerted inhibitory effects on cell migration and invasion in diverse cancer cell lines, including PC3 and DU145 prostate cancer cells [72], HCT116 and LoVo colorectal cancer cells [73], HeLa cervical cancer cell [97], SPC-A-1 and A549 lung cancer cells [55], A357, C8161, A375 and MV3 melanoma cells [58], MNNG/HOS and U2OS osteosarcoma cells [65], U251 glioblastoma cells [54], HepG2 hepatocellular carcinoma cells [50, 52], MDA-MB-231 cell, 4T1 and MCF-7 breast cancer cells [36, 42, 43].

Migration and invasion of tumor cells depend on the degradation of extracellular matrix by matrix metalloproteinases (MMPs) [98]. After treatment with lycorine, the protein levels of MMP2, MMP3, and MMP9 decreased [42, 43, 50, 65, 68]. lycorine suppressed migration and invasion by inhibiting the STAT3 signaling pathway in osteosarcoma cells and breast cancer cells, while also enhancing the expression of the negative STAT3 regulator, SHP-1 [43, 65]. Thus, the inhibition of the STAT3 pathway by lycorine in tumor cells can lead to various effects, including inducing apoptosis and suppressing migration and invasion. The inhibition of metastasis by lycorine and lycorine hydrochloride were associated with the suppression of the Src signaling pathway both in breast cancer [42] and cervical cancer [97]. In another study, lycorine inhibited migration of MDA-MB-231 and MCF-7 breast cancer cell by decreasing β-catenin and F-actin and increasing GSK-3β [36]. Additionally, lycorine activated Rho-associated coiled-coil containing protein kinase 1 (ROCK1), thereby suppressing the migration of HepG2 cells [50]. In C8161 and A375 cells, lycorine hydrochloride promoted the ubiquitin-mediated degradation of β‐catenin, thereby reducing downstream expression of pro-metastatic genes such as *Axin2* [68]. In CT26 lung metastasis model, lycorine hydrochloride treatment inhibited lung metastasis and upregulated DDIT3 and DR4 expression while downregulating β-catenin [46]. In lung cancer tissues, lycorine increased the protein level of Salvador homologue 1 (SAV1) by suppressing ubiquitin-mediated degradation and suppressed metastasis [55]. Strategies for treating metastatic tumors are more complex than those for primary tumors [92]. The observed inhibitory effect of lycorine and lycorine hydrochloride on tumor metastasis suggests promising avenues for cancer treatment, offering new possibilities to address the complexities associated with metastatic disease. To further validate these findings, more studies involving metastasis-related animal models should be conducted to explore the potential mechanisms and therapeutic implications of lycorine and lycorine hydrochloride in inhibiting cancer metastasis.

### Modulating immune system

The widespread application of immunotherapy has brought new hope to cancer treatment, with immune checkpoint inhibitors such as PD-1/PD-L1 antibodies serving as a prominent example [99]. Many natural products hold enormous potential in enhancing immunotherapy [100]. For example, nature-derived ginsenoside Rh2 improved anti-PD-L1 immunotherapy by reinvigorating CD8^+^ T cells by upregulating CXCL10 [101]. Additionally, blockage of cytotoxic T-lymphocyte associated protein 4 (CTLA-4) expressed on the surface of activated T cells improves T-cell immunity. Lycorine hydrochloride also showed the capacity to be applied in immunotherapy. In an orthotopic and metastatic renal cell carcinoma tumor-bearing mouse model, the combined treatment of lycorine hydrochloride and anti-CTLA-4 demonstrated a significant reduction in the ratio of CD4^+^ Foxp3^+^ T-regulatory cells, which plays an immunosuppressive role in TME. At the same time, lycorine hydrochloride treatment alone and in combination with anti-mouse CTLA-4 was also able to significantly increase the ratio of cytotoxic CD8^+^ T cells, signifying a notable enhancement in tumor immunity. In comparison to singular treatments with lycorine hydrochloride and anti-CTLA-4, the combination therapy exhibited better tumor inhibition effects [70]. These studies highlight the capacity of lycorine hydrochloride to modulate TME, suggesting their potential application in combination with immunotherapy to augment therapeutic outcomes. Although lycorine and lycorine hydrochloride show potential in enhancing immunotherapy, the precise mechanisms remain unclear. Further research is needed to determine whether they exert their effects by directly modulating immune cells or by altering tumor cells to affect the immune microenvironment.

## Direct binding targets

**Fig. 2:**
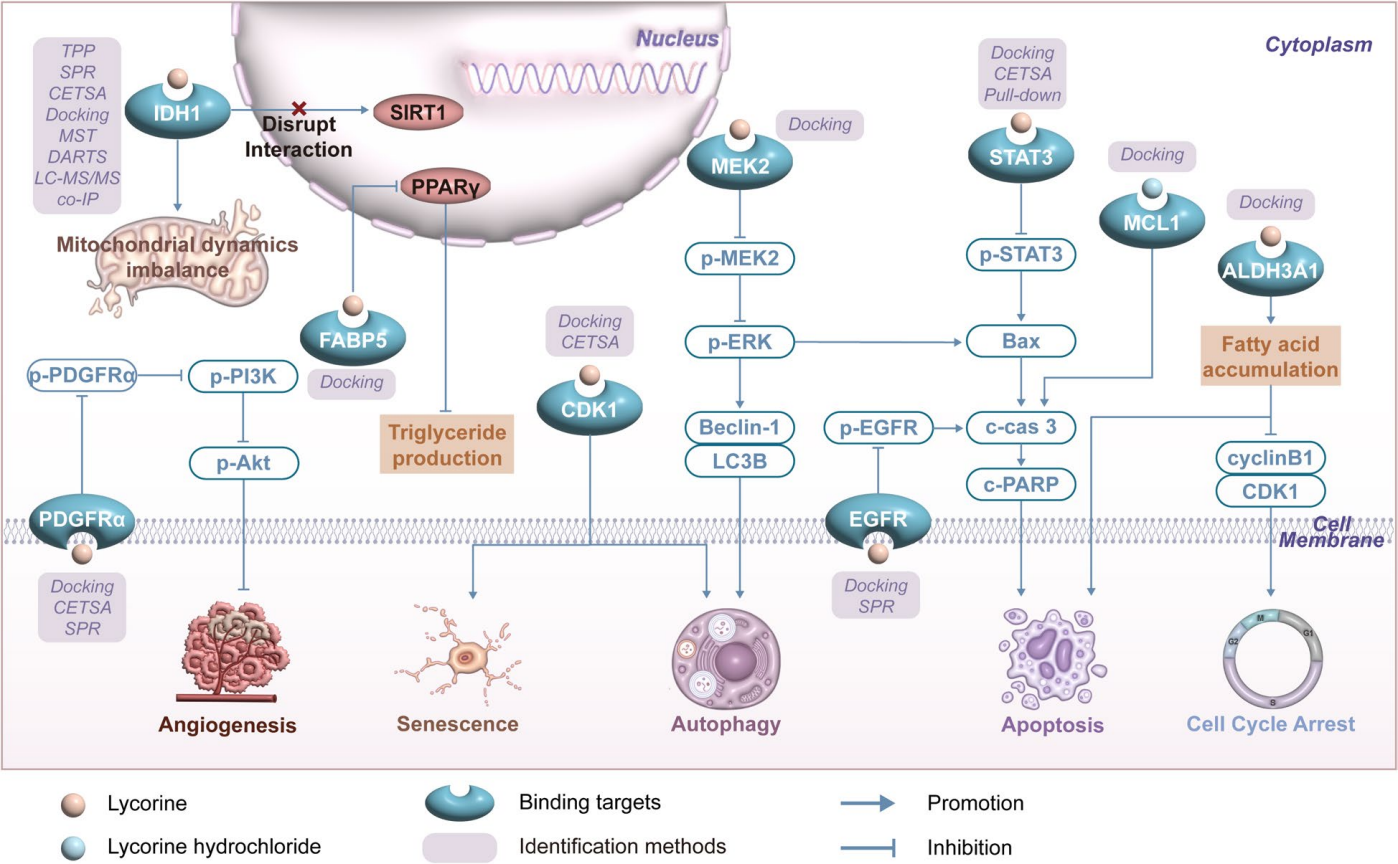
Schematic of the identified binding targets of lycorine in cancer cells. Lycorine and lycorine hydrochloride bind to distinct targets within cancer cells, subsequently modulating the downstream pathways and eliciting a spectrum of effects, including inhibiting angiogenesis, initiating cell senescence, inducing autophagy and apoptosis, prompting cell cycle arrest, and modulating metabolism

The anticancer effects of lycorine and lycorine hydrochloride have been studied for decades. Recent technological advancements have clarified their molecular targets, which is crucial for understanding their mechanisms of action. Existing research has identified potential binding targets of lycorine and lycorine hydrochloride (Fig. 2). For example, surface plasmon resonance (SPR), cellular thermal shift assay (CETSA), thermal proteome profiling (TPP) assays revealed that lycorine directly targeted the C-terminal domain of Isocitrate dehydrogenase 1 (IDH1), thereby disrupting its interaction with the deacetylase enzyme sirtuin 1 (SIRT1) and significantly triggering oxidative stress in HCT116 colorectal cancer cells [33]. In RKO and SW480 colorectal cancer cells, STAT3 was a target of lycorine confirmed by CETSA, pull-down assay, and molecular docking assays [102]. Moreover, lycorine exhibited a more potent anti-proliferation effect in glioblastoma multiforme cells with heightened expression of EGFR. SPR assays indicated that lycorine can bind to EGFR, consequently suppressing its phosphorylation [54]. In addition, CETSA and molecular docking assays confirmed spatial binding of lycorine to CDK1 and PDGFRα [78, 93]. Docking simulations have indicated potential interactions between lycorine or lycorine hydrochloride and various proteins, including FABP5 [81], aldehyde dehydrogenase 3A1 (ALDH3A1) [34], MEK2 [85], and MCL1 [47]. However, experimental validation is necessary to confirm these interactions, as evidence from docking alone may not be sufficient. Overall, lycorine and lycorine hydrochloride are likely to be multitargeted based on existing reports. This property may contribute to their efficacy in various types of cancer by binding to different targets and affecting multiple signaling pathways. As multitargeted agents, they hold considerable potential in mitigating drug resistance. For instance, in cases of target resistance mutations, lycorine and lycorine hydrochloride may retain their anticancer effects through alternate targets. In summary, further research into the exact molecular targets of will significantly advance their mechanism investigation and therapeutic potential.

## Pharmacokinetics

Enhanced comprehension of the anticancer potential of lycorine and lycorine hydrochloride necessitates further investigation of pharmacokinetic studies, considering the current research deficit. Generally, lycorine and lycorine hydrochloride demonstrated fast absorption kinetics across various studies, with T_max_ consistently less than 0.5 h [103–105]. Lycorine and lycorine hydrochloride exhibited rapid elimination from the plasma of rats and beagle doges, with mean plasma elimination half-life (t_1/2_) mostly less than 3 h [103, 105]. The oral bioavailability of lycorine hydrochloride at a dose of 1 mg/kg is approximately 40% [105]. Parameters such as area under the plasma concentration–time curve from 0 to infinity (AUC), mean residence time of drug in plasma (MRT), t_1/2_, time to reach maximum plasma concentration (T_max_), and maximum plasma concentration (C_max_) differed in many studies. Specific parameters are detailed in Table 3. These differences may be attributed to various factors, including differences in species, age, sex, and health status of the animals, dosage, and routes of the administration. Additionally, coadministration with other drugs influenced the pharmacokinetic properties of lycorine and lycorine hydrochloride, possibly due to drug-drug interactions. For instance, pretreatment of 0.2 mg/kg antiemetic drug ondansetron (OND) in beagle dogs by *i.v.* injection can alleviate nausea and emesis induced by lycorine hydrochloride and alter its pharmacokinetics [106]. Furthermore, following oral administration of *Lycoris radiata* at an oral dose of 628 mg/kg in rats, T_max_ of lycorine hydrochloride was longer compared to the administration of lycorine hydrochloride alone [107]. The changed pharmacokinetics properties implied potential interactions between lycorine or lycorine hydrochloride and other compounds, underscoring the need for further investigation into their combined effects on pharmacokinetics. Metabolomic assays conducted using beagle plasma and rat liver microsomes affirmed that the liver is the primary site of lycorine hydrochloride metabolism [31, 108]. Electrochemical metabolic simulation indicated that Phase I metabolism involved dehydrogenated lycorine, its hydroxy-derivative, and ungeremine [108]. Research on pharmacokinetics studies of lycorine and lycorine hydrochloride remains inadequate at the moment. More comprehensive pharmacokinetic studies are needed to thoroughly investigate the absorption, distribution, metabolism, and excretion of lycorine and lycorine hydrochloride. Understanding the pharmacokinetic characteristics is crucial for optimizing the therapeutic administration of lycorine and lycorine hydrochloride, with continuous dosing at safe levels potentially enhancing their anticancer efficacy. In summary, comprehensive pharmacokinetic studies are imperative for maximizing the therapeutic potential of lycorine and lycorine hydrochloride in cancer treatment.

**Table 3:** Pharmacokinetic parameters of lycorine and lycorine hydrochloride

Animal	Method	Dose	Administration route	AUC_(0-t)_ (ng/ml h)	MRT (h)	t_1/2_ (h)	T_max_ (h)	C_max_ (ng/ml)	Reference
Mouse	HPLC–MS	10 mg/kg lycorine	*i.p*	2418.17 ± 905.67	5.79 ± 1.44	5.58 ± 0.60	0.17	4730.00 ± 520.00	[104]
		10 mg/kg lycorine	*i.v*	3170.00 ± 668.67	2.92 ± 1.11	3.43 ± 0.78	0.08	5470.00 ± 680.00	
Rat	HPLC–MS/MS	5 mg/kg lycorine	*i.p*	2075.85 ± 347.78	2.38 ± 0.18	1.44 ± 0.57	0.25	1340.72 ± 123.55	[103]
		10 mg/kg lycorine	*i.p*	6083.59 ± 637.28	2.65 ± 0.25	3.15 ± 1.48	0.22 ± 0.05	3498.92 ± 326.03	
		20 mg/kg lycorine	*i.p*	12,901.46 ± 3957.43	2.88 ± 0.45	2.89 ± 0.63	0.25	6119.73 ± 879.73	
Rat	LC–MS/MS	628mg/kg *lycoris radiata* extract	*p.o*	2048.13 ± 198.49	7.87 ± 0.24	5.72 ± 1.00	2.83 ± 0.41	205.47 ± 24.47	[107]
Beagle dog	HPLC	1 mg/kg lycorine hydrochloride	*s.c*	3097.00 ± 291.00	–	0.66 ± 0.09	0.50 ± 0.00	2370.00 ± 335.00	[105]
		1 mg/kg lycorine hydrochloride	*i.v*	2489.00 ± 583.00	1.02 ± 0.20	0.51 ± 0.04	0.11 ± 0.03	5919.00 ± 382.00	
		1 mg/kg lycorine hydrochloride	*p.o*	986.00 ± 332.00	3.08 ± 1.50	1.04 ± 0.21	0.50 ± 0.14	494.00 ± 267.00	
Beagle dog	HPLC	2 mg/kg lycorine hydrochloride	*s.c*	6393.00 ± 124.00	1.28 ± 0.19	0.80 ± 0.11	0.50 ± 0.00	4545.00 ± 281.00	[106]
		0.2 mg/kg OND (pretreatment) and 2 mg/kg lycorine hydrochloride	OND *i.v*., lycorine hydrochloride *s.c*	10,270.00 ± 927.00	1.85 ± 0.28	1.35 ± 0.08	0.58 ± 0.08	6339.00 ± 283.00	

## Safety

Based on the existing limited studies, lycorine and lycorine hydrochloride exhibit low toxicity to normal cells and healthy mice [35, 42]. Following *i.p.* administration of lycorine hydrochloride, the LD_50_ was calculated to be 112.2 mg/kg based on the mortality observed within 3 days. However, when administered orally, the LD_50_ increased to 344 mg/kg [109], suggesting lower toxicity through gastrointestinal routes, possibly due to reduced absorption via the stomach. In vitro experiments further indicated that lycorine and lycorine hydrochloride exhibited minimal toxicity towards normal human cells, such as bone marrow stromal cells, highlighting their potential selectivity for targeting glioblastoma multiforme cells instead of normal brain tissue cells [54]. Transient emetic effects were observed following subcutaneous (*s.c.*) and *i.v.* injection of lycorine hydrochloride, generally subsiding within 2.5 h without affecting biochemical or hematological safety [105]. Lycorine less than dosage of 10 mg/kg by *i.p.* showed no significant adverse effects on the central nervous system of mice [30]. Furthermore, administration of lycorine and lycorine hydrochloride did not cause obvious side effects on body weight or overall health status in tumor-bearing mouse models [33, 40, 42, 46]. These findings indicate the promising safety properties of lycorine and lycorine hydrochloride in preclinical investigations, although long-term toxicity assessments of lycorine and lycorine hydrochloride are still lacking.

## Discussion

### Prospects of druggability

Lycorine and lycorine hydrochloride are promising candidates for clinical translation in anticancer therapy due to several key factors. (1) Potent anticancer effect: Numerous studies have demonstrated the excellent anticancer activity of lycorine and lycorine hydrochloride in various cancers in both in vitro and in vivo experiments. (2) Low toxicity: lycorine and lycorine hydrochloride demonstrate minimal toxicity, ensuring safety in clinical applications. (3) Potential for combination therapy: Enhanced anticancer efficacy has been observed when lycorine and lycorine hydrochloride were used in combination with other drugs, especially in alleviating drug resistance and impacting the TME. (4) Wide availability: lycorine’s abundant source from *Amaryllidaceae* plants and efficient synthesis approaches ensure a stable supply for both research and clinical applications [110, 111]. All these advances demonstrate that lycorine is a promising lead compound with a novel chemical structure, holding significant potential for clinical translation.

**Fig. 3:**
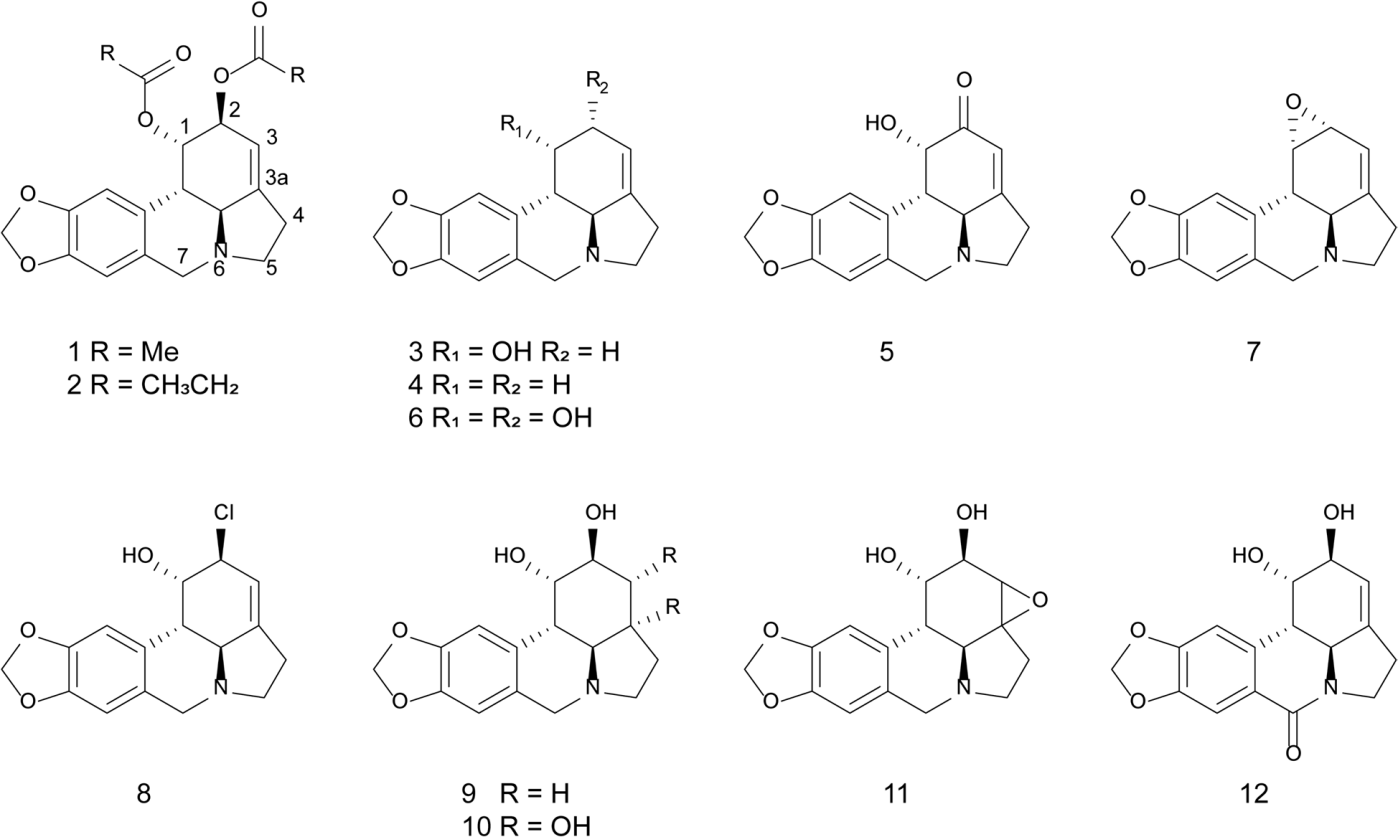
Some represented lycorine derivatives

Enhancing the anticancer activities of lycorine and lycorine hydrochloride through structural modification stands out as a promising approach to further improve their druggability. Various modifications of lycorine were conducted, and the structures of these derivatives are shown in Fig. 3. Notably, a lycorine derivative, 1, 2-di-O-allyllycorine, exhibited 100 times greater potency against U373 human glioblastoma cells than lycorine itself [112]. Based on the existing modifications of lycorine, the structure–activity relationship (SAR) was summarized to guide structural optimization. SAR study revealed some critical observations. 1) The presence of free hydroxyls at C-1 and C-2 and their stereochemistry have significant effects on the retention of bioactivity. The esterification and etherification of the hydroxyls at C-1 and C-2 in lycorine, such as compounds 1 and 2, resulted in the reduction of cytotoxic activities [16, 112–116]. Similarly, the removal or oxidation of the hydroxyl(s) at C-1 and/or C-2 (compounds 3, 4, and 5) also reduced cytotoxic activities. 2-Epi-lycorine (6), an epimer of lycorine only displayed weak cytotoxicity [116]. Interestingly, 1,2-R-epoxylycorine (7) and lycorine chlorohydrin (8) demonstrated cytotoxicity comparable to that of lycorine [113, 115], and their notable efficacy was likely attributable to their intracellular conversion to lycorine via nucleophilic reaction with water [115]. (2) The Δ^3,3a^ double bond in lycorine appeared to be essential for its antiproliferative activity. The reduction of Δ^3,3a^ double bond (9), along with dihydroxylation (10) and epoxidation (11) at the site, led to the loss of activity [112, 113, 115, 116]. (3) The basictiy of N-6 has an important role in the retention of cytotoxicity, as the oxidation of C-7 in lycorine to form an amide (12) resulted in the loss of activity [112, 115]. The SAR analysis clarified the active groups of lycorine, which not only guides subsequent structural modification research but may also offer valuable insights for target identification. Furthermore, structural modifications may enhance the selectivity and binding affinity of lycorine for specific molecular targets, thereby improving the efficacy.

However, a significant limitation in the druggability of lycorine and lycorine hydrochloride, which has often been overlooked, is their rapid elimination from plasma, potentially restricting their therapeutic efficacy. Current structural modifications have primarily focused on enhancing activity, but improvements in bioavailability and prolongation of circulation time through structural changes remain unexplored. This gap likely arises from the challenge of modifying pharmacokinetic properties while maintaining the active groups.

Alternative pharmaceutical approaches, such as sustained- and controlled-release formulations, offer potential strategies to extend in vivo circulation time and enhance efficacy [117]. Recent efforts in formulation development have been made. For example, mannosylated lipid nano-emulsions loaded with lycorine-oleic acid ionic complex demonstrated improved inhibitory effects and enhanced absorption on A549 cells [32]. However, it remains unclear whether this complex can effectively enhance in vivo efficacy. Furthermore, lycorine gold-based nanocomposites, designed to target tumor sites, have been shown to promote a high level of ROS production and exert efficacy in combination with photothermal therapy in the MNNG/HOS tumor-bearing mouse model [118]. Additionally, sulfobutyl-beta-cyclodextrin-based formulations of lycorine and lycorine hydrochloride achieved pH-sensitive drug release [119]. Despite these advancements, research on delivery systems for lycorine and lycorine hydrochloride remains insufficient. It is crucial to assess whether these formulations can effectively extend circulation time and enhance efficacy in animal models beyond cell-level experiments. The clinical application of existing formulations faces challenges, particularly in terms of controllability, stability, efficacy, and safety, indicating that there is still a long journey to explore. While Amaryllidaceae-derived compounds like Lycorine show potential for cancer therapy, rigorous clinical trials are necessary to establish their safety and efficacy before routine use. Addressing these issues holds promise for significantly improving the druggability of lycorine and lycorine hydrochloride.

### Insights into broader mechanism investigation

Lycorine and lycorine hydrochloride demonstrated anticancer effects through the regulation of multiple signaling pathways in tumor cells, including mTOR, NF-κB, JNK/STAT3, and ERK, among others. Previous research primarily focused on their direct impact on cancer cells, overlooking the TME due to technological constraints and limited understanding. TME is a complex system that, in addition to tumor cells, includes many types of immune cells, cancer-associated fibroblasts, endothelial cells, and others, playing a crucial role in cancer progression [120, 121]. As more understanding on cancer and immunotherapy gains prominence clinically, targeting the TME is increasingly considered a promising anticancer treatment strategy [122, 123]. Therefore, merely studying the effects of lycorine and lycorine hydrochloride solely on tumor cells is insufficient. Despite limited research on immunomodulatory effects within TME, lycorine was found to modulate T-cell metabolism by inhibiting ERK in cardiac allografts, indicating its potential regulatory effect on the immune system [124]. Burgeoning technologies such as single-cell omics [125], bioengineered 3D in vitro model systems [126], and so on, can be applied to detect their impact on TME.

Furthermore, modulating the microbiome is being used in treating various diseases, including cancer. There is increasing evidence suggesting that the microbiome may influence treatment outcomes [127]. To date, there have been no reports on whether lycorine and lycorine hydrochloride can modulate the microbiome during cancer treatment, representing an orientation for future research.

Additionally, tumor metabolism significantly promotes critical biological processes such as the proliferation, growth, migration, and invasion of cancer cells [128]. Regulating metabolic pathways in cancer cells is increasingly recognized as a feasible anticancer strategy [129]. However, the impact of lycorine and lycorine hydrochloride on cellular metabolism has not been adequately explored. Several recent studies have reported that lycorine can inhibit tumor cells by regulating lipid metabolism [34, 37, 81]. However, there have been no reports on the effects of lycorine and lycorine hydrochloride on other types of metabolism, such as glycolysis and amino acid metabolism. The rise of new technologies in recent years may aid in future research on the metabolic effects of lycorine and lycorine hydrochloride. For example, single-cell and spatial metabolomics can be used to assess changes in metabolic levels of important cell subpopulations; Integrating genomics and metabolomics can help explore the relationship between metabolic changes and tumor progression; Additionally, tracers and isotopes can be utilized for metabolic imaging [130]. Exploring the potential impact of lycorine and lycorine hydrochloride on the metabolism in the future could also provide valuable insights into their therapeutic mechanisms and optimize their clinical application.

### Inspiration from target identification

Identifying binding targets is essential for a better understanding of the therapeutic potential and possible side effects of lycorine and lycorine hydrochloride. In the studies on lycorine and lycorine hydrochloride, research on target identification remains quite limited. Among existing studies, IDH1 is likely a confirmed target of lycorine in colorectal cancer [33]. Based on the identified targets and various anticancer mechanisms, it is speculated that lycorine and lycorine hydrochloride exhibit multi-targeting properties. Therefore, in the process of target identification based on efficacy and phenotype, a comprehensive analysis of proteomic variations across different tumor types is essential to optimize target specificity and therapeutic efficacy. Furthermore, as the effects and mechanisms of lycorine and lycorine hydrochloride on other cells within the TME are unclear, the identification of targets also plays a crucial role in elucidating the mechanisms of action. This necessitates a broader exploration into the interactions between lycorine, lycorine hydrochloride and the TME, considering the complex impact on immune cells, the extracellular matrix, and others. Extensive experimental verification is still required, such as RNA sequencing, CETSA [131], SPR, stability of proteins from rates of oxidation (SPROX), drug affinity responsive target stability (DARTS) [132], affinity chromatography, selective isolation of proteins (SIP), TPP, pulldown assay [133], multiomics methods [134], and even many computational methods for prediction of drug-protein interactions [135, 136]. Additionally, the functional verification of target genes can be achieved through gene knockdown, knockout or overexpression experiments. The development of target identification can further improve the druggability of lycorine and lycorine hydrochloride.

## Conclusion

In conclusion, the collective findings from numerous studies have highlighted the remarkable anticancer effects of lycorine and lycorine hydrochloride through diverse mechanisms. Future studies should delve into understanding the in-depth mechanisms of lycorine and lycorine hydrochloride with the help of burgeoning new technologies, including their impact on TME, regulation of cellular metabolism, specific direct molecular targets, etc. Moreover, advancements aimed at enhancing their bioavailability and pharmacokinetic profiles will further bolster their potential as clinical anticancer therapeutics. In essence, lycorine and lycorine hydrochloride represent promising candidates for the development of novel cancer treatments, illuminating a path towards clinical applications.

## Data Availability

Not Applicable.

## Abbreviations

ALDH3A1: Aldehyde dehydrogenase 3A1
AUC: Area under the plasma concentration–time curve from 0 to infinity
BTZ: Bortezomib
c-cas 3: Cleaved caspase-3
c-cas 9: Cleaved caspase-9
CDKs: Cyclin-dependent kinases
CETSA: Cellular thermal shift assay
c-PARP: Cleaved PARP
C_max_: Maximum plasma concentration
DARTS: Drug affinity responsive target stability
FDA: U.S. Food and Drug Administration
*i.g.*: Oral gavage
*i.p.*: Intraperitoneal injection
*i.v.*: Intravenous injection
MMPs: Matrix metalloproteinases
OND: Ondansetron
PTX: Paclitaxel
RBPJ: Recombination signal binding protein for immunoglobulin kappa J region
ROS: Reactive oxygen species
ROCK1: Rho-associated coiled-coil containing protein kinase 1
SAR: Structure–activity relationship; SAV1, Salvador homologue 1
*s.c.*: Subcutaneous injection
SHP-1: SH2 domain-containing phosphatase 1
SIRT1: Sirtuin 1
SIP: Selective isolation of proteins
SPR: Surface plasmon resonance
SPROX: Stability of proteins from rates of oxidation
TME: Tumor microenvironment
T_max_: Time to reach maximum plasma concentration
TPP: Thermal proteome profiling
t_1/2_: Mean plasma elimination half-life
